# Recurrent ischemic stroke in a case of Takayasu’s arteritis, mimicking multiple sclerosis

**Published:** 2019-07-06

**Authors:** Payam Sasannejad, Mahdieh Verdipour, Mona Asadi, Hamideh Ahmadi

**Affiliations:** Department of Neurology, School of Medicine, Mashhad University of Medical Sciences, Mashhad, Iran

**Keywords:** Takayasu Arteritis, Multiple Sclerosis, Stroke, Granulomatous Angiitis, White Matter

Takayasu’s arteritis (TA) is a chronic granulomatous vasculitis with yet unknown etiology that affects the aorta and its main branches leading to progressive arterial stenosis. Neurovascular complications occur in less than 20% of patients with TA; however, they are a major source of morbidity in these patients.^1^ Low incidence of the disease, especially when presenting with atypical symptoms, can considerably delay the correct diagnosis and treatment. Attacks of focal neurologic deficits due to fluctuating brain ischemia may be difficult to distinguish from other neurologic disorders, especially relapsing and remitting multiple sclerosis, considering the demographic predominance of both disorders in young women. We present a 36-year-old woman referred to our department after the second attack of left hemiparesis. She experienced the first attack of left-sided weakness 4 months before. Examination at the time revealed left hemiparesis accompanied with left hemihypesthesia, upward left plantar response, and unremarkable general examination. She was apparently normal before these attacks, and reported no vascular risk factors. At the first admission, she was diagnosed with multiple sclerosis based on a brain magnetic resonance imaging (MRI) evidence of multiple foci of gadolinium-enhancing and non-enhancing white matter lesions in bilateral periventricular and subcortical areas (Figure 1, a and b), and negative workup for systemic vasculitis autoantibodies, anti-human immunodeficiency viruses (HIV) and human T-cell leukemia virus type 1 (HTLV-1) serum antibodies, and normal serum acetylcholine esterase and vitamin B12 levels. Cervical MRI was unremarkable. A gradual improvement was evident after treatment with a 5-day course of pulse methylprednisolone, and she was discharged on interferon beta.

At her second admission, she presented with another attack of left-sided weakness of grade 3 based on the Medical Research Council (MRC) scale accompanied with hemihypesthesia.

She also reported bilateral blurred vision. Fundoscopic examination was normal. Analysis of the cerebrospinal fluid (CSF) showed normal pattern with no oligoclonal bands, and visual evoked potentials showed bilateral normal waveform latencies. A second MRI showed an acute wedge-shaped lesion involving right parietal cortex with a restricted diffusion pattern suggestive of an ischemic infraction (Figure 1, c). On systemic examination, arterial pulses of upper extremities were nonpalpable at the right side and weak at the left. Blood pressure measured in the left arm was 80/60 mmHg, but it was unrecordable using routine devices in the right arm.

**Figure 1:**
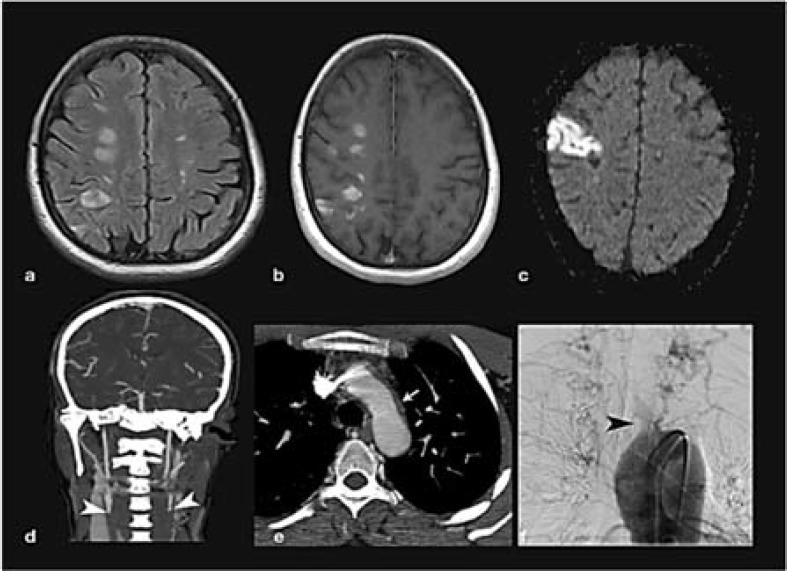
Imaging findings of the patient; magnetic resonance imaging (MRI) at the first attack, axial T2 fluid attenuated inversion recovery (a), and T1 post-contrast (b) sequences showing multiple foci of gadolinium enhancing and non-enhancing signal changes in the periventricular and subcortical white matter. Diffusion weighted MRI at the second attack (c) revealed an area of restricted diffusion in right parietal cortex indicative of an ischemic lesion. Coronal view computed tomography (CT) angiography of brain and neck at the second attack (d) showing occlusion of bilateral common carotid arteries (white arrowheads). Axial CT angiography at the level of aortic arc at the second attack (e) showing thickening of the aortic wall (arrow). Digital subtraction angiogram at the second attack (f) revealing complete occlusion of the main branches of the aortic arc (dark arrowhead).

A brain, neck, and aortic arc computed tomography (CT) angiography displayed a high-grade stenosis at the origin of the aortic main branches with more than 90% circumferential narrowing of bilateral common carotid and left subclavian arteries, and circumferential thickening of the aortic arc wall (Figure 1, d and e). Subsequent digital subtraction angiography (DSA) confirmed complete occlusion of the main branches of the aorta (Figure 1, f) compatible with TA type I.^2^ The diagnosis of TA was made according to the revised Ishikawa diagnostic criteria.^2^ Interestingly, tuberculosis DNA was reported positive on CSF sample of the patient using polymerase chain reaction (PCR) technique. There was no signs of active tuberculosis in the brain or other organs and inflammatory markers [erythrocyte sedimentation rate (ESR) and C-reactive protein (CRP)] were in normal range. Considering the absence of other specific features of tuberculous aortitis, such as aneurysm formation, erosion, or dissection of the vessel wall, and no signs of active tuberculosis in the central nervous system (CNS), lungs, or other organs, and normal inflammatory markers, we considered this finding a false positive. However, previous epidemiological and experimental studies suggested a possible causative role of tuberculosis in development of TA;^3^ hence we cannot exclude this possibility in our patient. Patient declined a second CSF sampling. Corticosteroid treatment was started with 1 mg/kg/day oral prednisolone which resulted in significant improvement. Treatment was continued with 50 mg oral prednisolone (planned for subsequent slow tapering) and antiplatelet therapy. On follow-up after two years, neurologic signs were stable, and she had no new ischemic attacks. Morning dizziness was her sole complaint.

TA commonly presents with constitutional symptoms such as fever, malaise, muscle pain, weight loss, anorexia, and claudication of the upper limbs. Evaluations might reveal typical findings of elevated ESR, progressive decline of blood pressure, and absence of peripheral pulses.^1^ Neurologic complications occur in more than half of the patients with TA, and include headaches, dizziness, transient visual obscuration, transient ischemic attacks, and stroke.^1^ Corticosteroids can induce remission and stabilize course in at least half of the patients.^4^ Our patient presented with a focal neurologic deficit with bilateral white matter lesions resembling multiple sclerosis in whom development of a second attack a few months later led to the correct diagnosis. Visual symptoms in this patient could be explained based on severe stenosis of the neck arteries leading to ischemia of the globes and the optic nerves. Complete long-segment stenosis of the carotid arteries and patent vertebral arteries preclude surgical treatment at the time in our patient.

## Notes:

**How to cite this article:** Sasannejad P, Verdipour M, Asadi M, Ahmadi H. Recurrent ischemic stroke in a case of Takayasu’s arteritis, mimicking multiple sclerosi**s**. Iran J Neurol 2019; 18(3): 145-7.
